# Supplementary material for: Barriers to accessing maternal health care services in the Chittagong Hill Tracts, Bangladesh: A qualitative descriptive study of Indigenous women’s experiences
Source: PLoS One. 2020 Aug 11;15(8):e0237002. doi: 10.1371/journal.pone.0237002 (PMC7419110; doi:10.1371/journal.pone.0237002)
Supplement: Attachment — Submitted filename: Comments on Bangladesh PLOS manuscript March 2020.docx [file pone.0237002.s001.docx]

**Comments on Manuscript: Indigenous women’s perspectives on accessing maternal health care services in the Chittagong Hill Tracts (CHT), Bangladesh: a qualitative descriptive study**

Thank you for requesting me to review this manuscript. This paper is an important contribution to the use of MCH services and disrespect and abuse of women in MCH services by health workers. A lot of this work has been done in low (e.g. Malawi) and middle income countries. However, this paper is important because it focuses on marginalized communities.

**Introduction**

Perhaps it would be proper to indicate how many ANC visits WHO expects women to have.

**Analysis**

1. Was the analysis theory or data driven?

2. The analysis could benefit from further categorization or classification of the barriers according to various themes (e.g. health care barriers, individual barriers, community barriers etc.). The Bradley paper below could perhaps give some insights on how to arrange the themes.

A) **System barriers -** could look at those barriers related to the health care systems
B) **Health provider barriers** e.g. unethical practices that are done without informed consent of the participants who have a loop inserted without their knowledge, midwives and cleaners who request for payment from patients
C) **Individual barriers -** These would pertain to the barriers pertinent to the participants like lack of money, lack of knowledge etc.

Some categories were not necessarily barriers, but merits of the home care delivery, so maybe they could fall under perceptions of participants, like, the comparison they make between health care and home delivery.

I would add to line 655 - unethical and unfair demands

**References**

Bradley, S., McCourt, C., Rayment, J., & Parmar, D. (2016). Disrespectful intrapartum care during facility-based delivery in sub-Saharan Africa: a qualitative systematic review and thematic synthesis of women's perceptions and experiences. *Social Science & Medicine*, *169*, 157-170.
