# Supplementary material for: Barriers to accessing maternal health care services in the Chittagong Hill Tracts, Bangladesh: A qualitative descriptive study of Indigenous women’s experiences
Source: PLoS One. 2020 Aug 11;15(8):e0237002. doi: 10.1371/journal.pone.0237002 (PMC7419110; doi:10.1371/journal.pone.0237002)
Supplement: Attachment — Submitted filename: PONE-D-20-03726_reviewer Bangladeash qual access 2020 RM.docx [file pone.0237002.s002.docx]

| **Abstract** |  | **Suggestion** |
| --- | --- | --- |
| Line 45 (49-51) | States Bangladesh has successfully achieved the millennium goals but in Line -line 49 – 51 it is states Bangladesh is working towards this and that it looks promising to achieving the goal | As these statements contradict each other, please make one clear statement |
| Iine 47 | Not disbursed across all populations | This statement here lets the reader to believe that the indigenous people group was deliberately left out everywhere. ‘not disbursed across all population’. It appears the research question is about how indigenous women access the services, which indicates that maybe geographically the services were available, but women were not accessing them. Please re-write for clarity |
| Line 58 | Recruit a diverse a group of women | Please remove extra ‘a’.  What is meant with diverse, please clarify, do you mean age? as ethnicity is identified already the ethnicity Chakma, Marma and Tripura |
| Line 69 | Systematic | Is systemic meant here? |
| **Abstract conclusion** |  | What about information and services being available in women’s first language? |
| **Introduction**  Line 77-78 | Low-income country | In the abstract it states correctly low- and middle-income countries  Policy Brief (2018) by Debapriya Bhattacharya and Sarah Sabin Khan (copy available on request) |
| Line 85 | accessibility and  utilisations for cultural minority groups in Bangladesh, including Indigenous women | It would be useful to include a short paragraph explaining the difference between minority groups and indigenous groups and describe the general indigenous people group of Bangladesh. |
| Line 89 | eleven Indigenous communities | How where the three people group chosen out of the eleven? |
| Line 92 - 94 | While national MHC access rates have improved, the health system has not necessarily provided  inclusive or equitable care for women from diverse socio-cultural backgrounds | it is unclear how this statement relates to the indigenous people group of study. It appears the author assumes being indigenous means diverse socio-cultural backgrounds |
| Line 99 and 102 | 37% and 43% for national average | Which one is correct? |
| Line 109 | Tribal health | Does tribal equal indigenous? |
| Line 129 | Barriers | Why not enabler explorations as well? If women identify what works well then services would be able to focus on this too |
|  | A well written article of the study relating to the current study for publication. It suggested to enlist the support of the co-authors of that study to address the long sentences and repeat expressions in this article. | Akter S, Rich JL, Davies K, Inder KJ. Access to maternal health care services among Indigenous women in the Chittagong Hill Tracts, Bangladesh: A cross-sectional study. BMJ Open. 2019. doi: <http://dx.doi.org/10.1136/bmjopen-2019-033224> |
| Throughout medical language used | Delivery  Foetus  patients | Birth  Baby  women |
| Line 143 to Line 145 |  | Long sentence loses its clarity. |
| Line 195 | Ethics approval | Please state the approval numbers |
| Line 197 | privacy and confidentiality were maintained | How? |
| Table 1 | Under Chakma - Nearest health facilities & distance #5 UHC and District hospital  10-25 | 10-25 ?km ?m, descriptor is missing.  Table 1 could have benefitted to include gravida/parity to identify easily which women had more than one pregnancy |
| Line 214 and in Table 1 under ANC | The remaining five accessed private facilities which brings it to a total of 11 women | Further clarity around the 5 women who accessed private care but are listed as ‘0’ ANC |
| Throughout the article | upazilla health complex | Should that not be written with a capital? It is a noun/name? ‘Upazilla’ |
| Line 585 | child | Baby or newborn |
| Line 624 to 627 | Long sentence | Loses its clarity |
| Line 673 | Countries… | Plural, but only one reference for one country (Mexico) is provided |
| Line 710 to 713 | Long sentence | Loses its clarity |
| Line 718 | Strengths and limitations | It needs to be mentioned that the numbers in this study is small and therefore not necessarily generalisable. |
| Conclusion  Line 745 to 749 |  | Be mindful of generalisation to other indigenous communities. The data is only from a small sample of CHT women. |
| Acknowledgements  Line 761 | local community leaders | These were not mentioned in the article. What was their role? It may be appropriate to mention this in the article |
