# Supplementary material for: Barriers to accessing maternal health care services in the Chittagong Hill Tracts, Bangladesh: A qualitative descriptive study of Indigenous women’s experiences
Source: PLoS One. 2020 Aug 11;15(8):e0237002. doi: 10.1371/journal.pone.0237002 (PMC7419110; doi:10.1371/journal.pone.0237002)
Supplement: Attachment — Submitted filename: Response to Reviewers comments.docx [file pone.0237002.s003.docx]

The authors thank the Editor and Reviewers for their valuable comments to improve the quality of this manuscript. We have incorporated all of the comments, and the additions can be seen as track changes (red colour). Please see below for detailed response to reviewer’s comments.

| **Reviewer#1** | **Authors’ response** |
| --- | --- |
| **Comment #1**  Thank you for requesting me to review this manuscript. This paper is an important contribution to the use of MCH services and disrespect and abuse of women in MCH services by health workers. A lot of this work has been done in low (e.g. Malawi) and middle income countries. However, this paper is important because it focuses on marginalized communities. | The authors thank the Reviewer #1 for recognising the importance of the current manuscript on maternal health care services access among Indigenous women in the Chittagong Hill Tracts. As the key focus of this manuscript is on Indigenous women (i.e. marginalised communities), we have cited original research and reviews on Indigenous or ethnic women in low and middle-income countries. |
| **Comment #2**  Was the analysis theory or data driven? | The analysis was data driven. This is now stated in the Data analysis section of the Methods. Please, see line# 241; page: 11 |
| **Comment #3:**  The analysis could benefit from further categorization or classification of the barriers according to various themes (e.g. health care barriers, individual barriers, community barriers etc.). The Bradley paper below could perhaps give some insights on how to arrange the themes.   A) **System barriers -** could look at those barriers related to the health care systems B) **Health provider barriers** e.g. unethical practices that are done without informed consent of the participants who have a loop inserted without their knowledge, midwives and cleaners who request for payment from patients C) **Individual barriers -** These would pertain to the barriers pertinent to the participants like lack of money, lack of knowledge etc.   Some categories were not necessarily barriers, but merits of the home care delivery, so maybe they could fall under perceptions of participants, like, the comparison they make between health care and home delivery.  **References**  Bradley, S., McCourt, C., Rayment, J., & Parmar, D. (2016). Disrespectful intrapartum care during facility-based delivery in sub-Saharan Africa: a qualitative systematic review and thematic synthesis of women's perceptions and experiences. *Social Science & Medicine*, *169*, 157-170. | After careful reading of the recommended systematic review and meta-synthesis written by Bradley and colleagues (2016), the authors have agreed with the suggestion and revised the **Results** section. The current **Result** section has the following categorisations (Line # 321 – 730)  **MHC services are for dealing with complications:** “Why would I go, if I did not have any complications?”  **Barriers that prevented women from accessing MHC access**   - Lack of knowledge about the services and facilities - Lack of knowledge about the importance of attending ANC and PNC services - Distance and lack of available transport - Fears related to medical intervention     **Deterrents to future MHC service access**   - Lack of trust in quality of care at public facilities - Unofficial costs – even in so-called “free” facilities - Maltreatment from hospital staff - Lack of appropriate and accessible information   **Maternal health care values**   - The importance of home - Flexible payment options: “We can pay them (TBAs) later - Personal connections   Given that Indigenous women in the Chittagong Hill Tracts (CHT) have limited knowledge about, and access to, maternal health care services, they did not provide sufficient information regarding system barriers, health provider barriers and individual barriers. Therefore, the data would not fit well under categorisations suggested by the **Reviewer#1**. |
| **Comment #4:**  I would add to line 655 - unethical and unfair demands | Line 655 (new line # 815; page: 38) is revised as per suggestion: “unethical and unfair demands” added in track changes. |

| **Reviewer#2** | | | **Authors’ response** |
| --- | --- | --- | --- |
| **Abstract** |  | **Suggestion** |  |
| Line 45 (49-51) | States Bangladesh has successfully achieved the millennium goals but in Line -line 49 – 51 it is states Bangladesh is working towards this and that it looks promising to achieving the goal | As these statements contradict each other, please make one clear statement | The Authors thank Reviewer#2 for the valuable comments. The authors carefully checked lines between 49 and 51. The authors have revised the “Background” section into following:  *“Increased maternal health care (MHC) service utilisation in Bangladesh over the past decades has contributed to improvements in maternal health outcomes nationally, yet there is little understanding of Indigenous women’s experiences of accessing MHC services in Bangladesh.”* [new line# in revised document: 53-58; page: 3] |
| Line 47 | Not disbursed across all populations | This statement here lets the reader to believe that the indigenous people group was deliberately left out everywhere. ‘not disbursed across all population’. It appears the research question is about how indigenous women access the services, which indicates that maybe geographically the services were available, but women were not accessing them. Please re-write for clarity | The authors have revised the “Background” section into following:  *“Increased maternal health care (MHC) service utilisation in Bangladesh over the past decades has contributed to improvements in maternal health outcomes nationally, yet there is little understanding of Indigenous women’s experiences of accessing MHC services in Bangladesh.”* [Please, see in revised document: line# 53-58; page: 3] |
| Line 58 | Recruit a diverse a group of women | Please remove extra ‘a’.  What is meant with diverse, please clarify, do you mean age? as ethnicity is identified already the ethnicity Chakma, Marma and Tripura | Removed.  Statement changed to:  *Purposive sampling was used to recruit women representative of the population distribution in terms of age, ethnic community and service use experience.*  Please, see line # 63-64; page: 3 in the revised document. |
| Line 69 | Systematic | Is systemic meant here? | Corrected. Please, see line # 75-81; page: 4 |
| **Abstract conclusion** |  | What about information and services being available in women’s first language? | Abstract conclusion is revised as following:  “*Findings suggest that access to MHC service for the CHT Indigenous women may be improved through better health education in women’s first language, increased awareness of local services by Indigenous women and systemic reform*”.  Please, see line # 84-86; page: 4 |
| **Introduction**  Line 77-78 | Low-income country | In the abstract it states correctly low- and middle-income countries  Policy Brief (2018) by Debapriya Bhattacharya and Sarah Sabin Khan (copy available on request) | Revised and added “low and middle-income country”. Please, see new line# 89-90; page: 5 in the revised document. |
| Line 85 | accessibility and  utilisations for cultural minority groups in Bangladesh, including Indigenous women | It would be useful to include a short paragraph explaining the difference between minority groups and indigenous groups and describe the general indigenous people group of Bangladesh. | A short paragraph added explaining cultural minority groups and indigenous groups of Bangladesh under “Introduction”.  Please, see paragraph 2; line 100-114; page: 5-6 |
| Line 89 | eleven Indigenous communities | How where the three people group chosen out of the eleven? | The Chittagong Hill Tracts (CHT) consists of three hill districts: Khagrachhari, Rangamati and Bandarban. Eleven ethnic groups reside in these three districts. The Chakma, Marma and Tripura- groups live in all three districts, and they are prominent among the eleven Indigenous groups. Considering the population size of these groups and availability in the three districts, these three groups were chosen. |
| Line 92 - 94 | While national MHC access rates have improved, the health system has not necessarily provided  inclusive or equitable care for women from diverse socio-cultural backgrounds | it is unclear how this statement relates to the indigenous people group of study. It appears the author assumes being indigenous means diverse socio-cultural backgrounds | The sentence has been revised to clarify the information. Please, see under “Introduction” ; paragraph: 3; line# 120-126; page- 6 in the revised document. |
| Line 99 and 102 | 37% and 43% for national average | Which one is correct? | Both are correct.  37% is the national average for accessing facility delivery services.  43% is the national average of accessing all three MHC services (antenatal, facility delivery and postnatal) care services.  The sentence has been modified to clarify this.  Please, see line# 131-136; page: 6 in the revised document. |
| Line 109 | Tribal health | Does tribal equal indigenous? | “Tribal” is the term used in the constitution and health policy. However, these ethnic groups have been protesting against such term as it indicates their lower social status. This has been stated under “**Introduction**”; paragraph: 2; line# 100-114; page: 5-6 in the revised document |
| Line 129 | Barriers | Why not enabler explorations as well? If women identify what works well then services would be able to focus on this too | The participants in the interviews focused their discussions on barriers, reflecting their limited experiences of, and reluctance to access, MHC. However, a section exploring the factors that women value most in MHC has been added. This section explains why women prefer home birth, offering the types of lessons that may be transferred to health services and facilities to ‘enable’ more access in the future. The authors have revised the **Result** section under following categorisations: (Line # 321 – 730; page: 18-34)  **MHC services are for dealing with complications:** “Why would I go, if I did not have any complications?”  **Barriers that prevented women from accessing MHC access**   - Lack of knowledge about the services and facilities - Lack of knowledge about the importance of attending ANC and PNC services - Distance and lack of available transport - Fears related to medical intervention     **Challenges experienced that deterred from future MHC service access**   - Lack of trust in quality of care at public facilities - Unofficial costs – even in so-called “free” facilities - Maltreatment from hospital staff - Lack of appropriate and accessible information   **What Indigenous women of the CHT value in maternal health care**   - The importance of home - Flexible payment options: “We can pay them (TBAs) later - Personal connections |
|  | A well written article of the study relating to the current study for publication. It suggested to enlist the support of the co-authors of that study to address the long sentences and repeat expressions in this article. | Akter S, Rich JL, Davies K, Inder KJ. Access to maternal health care services among Indigenous women in the Chittagong Hill Tracts, Bangladesh: A cross-sectional study. BMJ Open. 2019. doi: <http://dx.doi.org/10.1136/bmjopen-2019-033224> | Thank you, the manuscript has been revised to address the long sentences. |
| Throughout medical language used | Delivery  Foetus  patients | Birth  Baby  women | Revised  “Foetus” into baby”  “Patients” into “women”  “Delivery” into Birth or Childbirth except where delivery is in relation to delivery facilities |
| Line 143 to Line 145 |  | Long sentence loses its clarity. | Revised. Please, under “Study setting”; Line# 182-185 page: 8.  *“The Khagrachhari district is hilly with a significant proportion of forested land* *[40]*. *This district was purposively selected because a range of public, private and non-profit health services were available to Indigenous women residing in the area, and because data on the Indigenous population were available [39,41].”* |
| Line 195 | Ethics approval | Please state the approval numbers | Revised. Please, see new line# 249; page: 11 in the revised document. |
| Line 197 | privacy and confidentiality were maintained | How? | Thank you, the following has been added:  “*Participation was voluntary, and confidentiality was maintained by using a pseudonym for interview participants. Results identify participants by a number only. Prior to accessing each para (village) for data collection, the interviewer contacted local community leaders of the Indigenous communities and sought their permission*.”  Please, see line# 250-254; page: 11 in the revised document. |
| Table 1 | Under Chakma - Nearest health facilities & distance #5 UHC and District hospital  10-25 | 10-25 ?km ?m, descriptor is missing.  Table 1 could have benefitted to include gravida/parity to identify easily which women had more than one pregnancy | Corrected to 10-25 km.  Regarding gravida/parity, an extra column has been added reporting the number of total pregnancy in both Table 1 and Table 2. Thank you. |
| Line 214 and in Table 1 under ANC | The remaining five accessed private facilities which brings it to a total of 11 women | Further clarity around the 5 women who accessed private care but are listed as ‘0’ ANC | The sentence has been clarified in the text line xx: *The remaining five accessed private facilities for either ANC (two women) or facility delivery services (three women).*  Please, see line# 271-272; page: 12 in the revised document. |
| Throughout the article | upazilla health complex | Should that not be written with a capital? It is a noun/name? ‘Upazilla’ | Corrected to “Upazila” after careful review of government documents on public administration. |
| Line 585 | child | Baby or newborn | All “child” terms have been changed to “baby or new-born where applicable. |
| Line 624 to 627 | Long sentence | Loses its clarity | Revised.  Please, see under Discussion; paragraph: 2:  line # 779-783. Page: 36 in the revised document. |
| Line 673 | Countries… | Plural, but only one reference for one country (Mexico) is provided | Another reference added.  Please, see line# 835; page: 39 in the revised document. |
| Line 710 to 713 | Long sentence | Loses its clarity | Revised into the following.  *Intercultural policies and interventions that support human rights and gender equity are needed to access hard-to-reach population groups such as the Indigenous women living in the CHT.*  Please, see paragraph: 10; line# 875-878. Page: 40 in the revised document |
| Line 718 | Strengths and limitations | It needs to be mentioned that the numbers in this study is small and therefore not necessarily generalisable. | The authors have revised the “Strengths and Limitations” section. Please, see Line # 883-909: page: 41-42 Please, see paragraph: 10; line# 874-877. Page: 40 in the revised document |
| Conclusion  Line 745 to 749 |  | Be mindful of generalisation to other indigenous communities. The data is only from a small sample of CHT women. | The authors have revised the “Conclusion” section. Please, see Line # 912-926: page: 42 |
| Acknowledgements  Line 761 | local community leaders | These were not mentioned in the article. What was their role? It may be appropriate to mention this in the article | Local community leaders gave informal approval to access the communities and conduct the fieldwork.  The authors have added a sentence addressing local community leaders’ roles under “Ethics” section.  Please, see line# 252-254; page: 11.  The authors have also revised the “Acknowledgement” section in track change. Please, see line# 940; page 43 in the revised document. |
