# Supplementary material for: Barriers to accessing maternal health care services in the Chittagong Hill Tracts, Bangladesh: A qualitative descriptive study of Indigenous women’s experiences
Source: PLoS One. 2020 Aug 11;15(8):e0237002. doi: 10.1371/journal.pone.0237002 (PMC7419110; doi:10.1371/journal.pone.0237002)
Supplement: Attachment — Submitted filename: Response to Reviewers comments.docx [file pone.0237002.s004.docx]

**PONE-D-20-03726R1: Akter et al. Barriers and enablers to accessing maternal health care services in the Chittagong Hill Tracts, Bangladesh: A qualitative descriptive study of Indigenous women’s experiences**

The authors thank the Editor and Reviewers for their valuable comments to improve the quality of this manuscript. We have incorporated all of the comments, and the additions can be seen as track changes (red colour). Please see below for detailed response to reviewer’s comments.

| **Reviewer#1** | **Authors’ response** |
| --- | --- |
| **Comment #1**  Most of the comments have been addressed. The last sentence can be shortened to read: Although there were two questions meant to elicit enablers of health service use, the data were limited as women focused more on barriers to access. | The authors thank the Reviewer #1 for the comments. |
| **Comment #2**  On page 4 line 97 there is an APA reference which should be corrected to align with the journal referencing. | The suggested reference has been changed to PloS One’s required referencing style. |
| **Comment #3**  There is a minor editorial on page 27 line 561, delete a. | Amended (in track change). |

| **Reviewer#2** | **Authors’ response** |
| --- | --- |
| **Comment #1**  It is commendable that the author has addressed most of the review feedback provided. Here are further recommendations to strengthen the article: - although it was recommended that the title changes to barrier and enablers..., there however are no enablers identified in the re-submitted article as suggested, hence I recommend now to remove the word 'enabler' from the title - line 148 remove the word facilitators, as they are not identified in the article - line 168 'upazilas' needs a capital U - line 242 please add women after 'Most (n=11)' | The Authors thank the Reviewer#2 the comments.  The title has now changed to  “**Barriers to accessing maternal health care services in the Chittagong Hill Tracts, Bangladesh: A qualitative descriptive study of Indigenous women’s experiences”**  All the suggested changes have been addressed in the revised document in track change. |
| **Comment #2**  The abstract conclusion and the article conclusion do not really reflect the findings. 'What is meant with 'systemic reform' ?- Revisit the following article and [https://bmjopen.bmj.com/content/9/10/e033224](https://protect-au.mimecast.com/s/D2-1CVARWBc0P80zfJjnBl?domain=bmjopen.bmj.com) and read the conclusions and abstract. It is informative to see that the language concern is being mentioned in the abstract and this additions is welcomed and importunate, however, it features only marginally in the study, hence other significant findings need to be mentioned e.g. fear, distance, costs, lack of trust, maltreatment from staff, lack of knowledge all are important barriers | The authors carefully have checked and revised the Conclusion under Abstract:  *“Improving access to MHC services for the CHT Indigenous women requires improved understandings of cultural values, priorities and concerns. Multifaceted reform is needed at individual, community and health systems levels to offer culturally appropriate health education and flexible service delivery options.”*  The authors also have revised Conclusion in the main manuscript. Please see Lines: 842-849 in page: 39 |
| **Comment #3**  Please also note on the reference list: reference 38 and reference 39 are missing their doi's. Either add them or delete doi | DOIs’ for those to references has been added. |
| **Comment #4**  Table 1 - #3 and #4 while km has now been added there needs to be gap between the number and km as it has been presented with all others, consistency is required. Thank you. | Amended as suggested in Tables 1 and 2. |
